# Monitoring the cognitive load in competitive environments in professional women’s basketball

**DOI:** 10.3389/fpsyg.2025.1523915

**Published:** 2025-04-23

**Authors:** Joan Fuster, Lluis Capdevila, Toni Caparros

**Affiliations:** ^1^Institut Nacional d’Educació Física de Catalunya (INEFC), Barcelona, Spain; ^2^Sports Research Institute, Universitat Autònoma de Barcelona (UAB), Bellaterra, Spain; ^3^Department of Basic Psychology, Universitat Autònoma de Barcelona (UAB), Bellaterra, Spain

**Keywords:** cognitive resources, team sports, load monitoring, integral training, training demands

## Abstract

This study aims to describe the dynamics of training loads during specific training sessions, to determine the possible differences among the metrics of Cognitive Load (CL), External Load (EL) and Internal Load (IL) between training sessions and to assess the possible relationship between the CL, EL and IL variables to completely monitor the athletes’ performance level. Ten professional female basketball players (age 26.45 ± 3.5 years) took part in this descriptive study throughout the second round of competition, completing a total of 11 competitive microcycles. The training sessions were classified according to the distance between the previous game and the next one (MD +/− X), making distinctions between MD + 2, MD-4, MD-3, MD-2 and MD-1. The following descriptive variables of the tasks were recorded: specificity, number of players, playing space, time pressure, decision-making and competitive stimulus. The analyzed variables were rate of perceived cognitive exertion (RPE Cog) and heart rate variability (HRV) for CL, total amount of high intensity actions (HI-T) and total sum of accelerations – decelerations (AD-T) for EL, and rate of perceived exertion (RPE) and summated heart rate zones (SHRZ) for IL. The load dynamics showed an increase in uncertainty throughout the microcycle, progressing from less to more specific, and a load distribution in which MD + 2 and MD-1 show the lowest values and MD-4, MD-3 and MD-2 the highest. Significant differences (*p* < 0.01) were found between sessions for all the analyzed variables. Possible relationships between the CL, EL and IL metrics were also established. This study shows the reality of a professional team, where the distance from the next match determines the dynamics of the workload, promoting an increase in uncertainty and specificity throughout the microcycle, thus causing an increase in cognitive load.

## Introduction

Basketball is an intermittent indoor team sport, in which many high intensity neuromuscular efforts, such as changes of direction, accelerations, decelerations and jumps, are performed between rest periods (Torres-Ronda et al., 2016). When a series of actions of a certain intensity are chained during the game, these periods are defined as the Most Demanding Scenarios (MDS) (Vázquez-Guerrero et al., 2020). The MDS fluctuate throughout games and vary between playing positions (García et al., 2022b). Due to the demanding and changing nature of basketball, monitoring the training load faced by players is a necessary process to optimize performance (Sansone et al., 2020). The training load has been defined as the input variable that is manipulated to obtain a desired response to training (Impellizzeri et al., 2019). This load has been described as cognitive load (CL), external load (EL) and internal load (IL) (Fuster et al., 2021). CL is the volitional allocation of mental resources to respond to the demands imposed by the received stimulus (Cárdenas et al., 2015). Due to this condition, several authors point out that Cognitive Load is related to the emotional state (Camacho et al., 2021; Cárdenas-Vélez et al., 2013). EL is the external stimulus applied to the athlete that is measured independently of its internal characteristics; and IL is the individual physiological response to the assumed stimulus (Soligard et al., 2016). These demands are accepted as separate constructs that must be analyzed and interpreted in the same context to achieve a better understanding of the adaptations that take place during sportive activities (Fuster et al., 2021).

Moreover, these training loads are distributed and organized in phases and cycles to promote an optimal condition for competition. (Nacleiro et al., 2013). In team sports, the microcycle is identified as the most important planning unit, understanding the microcycle not as the training week, but as the time between one game and the next (Tarragó et al., 2019) and will vary depending on the competitive calendar, having a direct effect on the training load (Oliva-Lozano et al., 2021b). In basketball, planning must be adjusted microcycle to microcycle depending on how the load is handled by the players (Piedra et al., 2021). The current trend in load monitoring research leans towards multivariate approaches that, through a more holistic lens, offer a greater insight into the nature of the dose–response (Weaving et al., 2017; Piedra et al., 2021; Fuster et al., 2021). Several investigations have reported on physical demands (EL) (Caparrós et al., 2018; Svilar et al., 2019; Vázquez-Guerrero and Garcia, 2021) or physiological demands (IL) (Torres-Ronda et al., 2016; Scanlan et al., 2017; Piedra et al., 2020) during basketball training and match cycles. However, to date, the available research on cognitive demands is limited (Perrey, 2022), as are longitudinal studies on load dynamics during the competitive period in women’s basketball (Power et al., 2022).

In this context, and considering the applicability and implications for load prescription in its three dimensions (CL, EL and IL), the performance optimization and injury prevention, this paper has three goals: (i) To describe the dynamics of training loads in order to monitor the performance level of the athletes in a specific training session; (ii) to determine the possible differences among the CL, EL and IL metrics between training sessions; and (iii) to assess the possible relationship between the CL, EL and IL metrics.

## Materials and methods

### Participants

Ten female professional basketball players (age, 26.45 ± 3.5 years; height, 178.82 ± 8.27 cm; weight, 75.3 ± 11.12 kg) participated in the study. The inclusion criteria for their participation required that they were part of the professional team roster during the 22/23 season. The exclusion criteria were to be involved in a rehabilitation process and not to completed more than 90% of the team’s total training sessions. During the study, 1 of the 11 players was unable to meet the inclusion criteria (due to injury). All players were informed of the risks and benefits of taking part in the study and gave written consent to participate in it. They were also allowed to decline the inclusion of their data. The study was conducted according to the Local Ethics Commission for Human Experimentation (protocol code CEEAH-5745).

### Procedure

A retrospective descriptive observational study was conducted in a Spanish women’s professional basketball team (LF Challenge) for a period of 11 weeks, within the second phase of the 2022–2023 competitive season.

**Figure 1:**
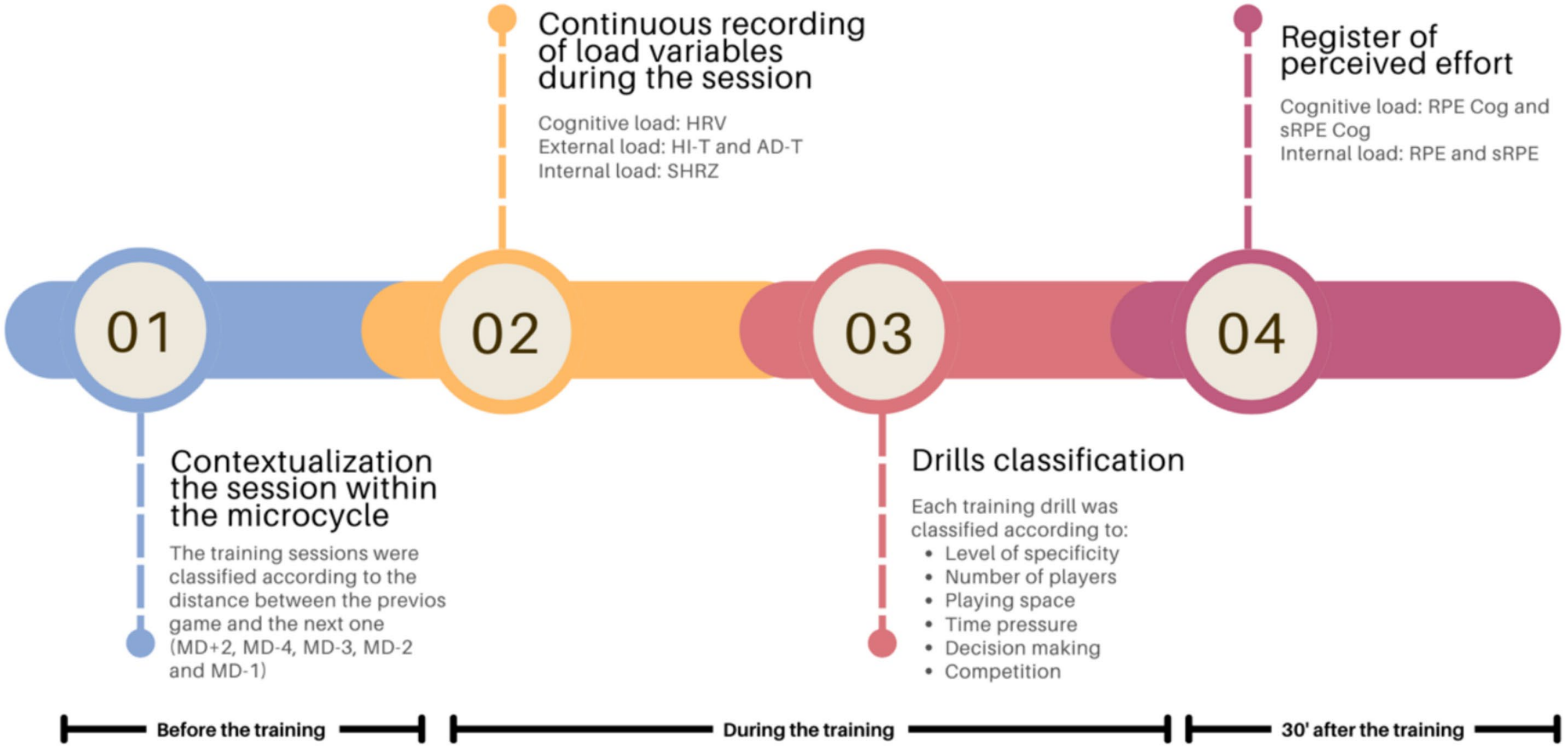
Summary diagram of the registration procedure. MD ± x: x days after (+) or before (−) the match day; HRV: RMSSD parameter of heart rate variability; HI-T: sum of actions at high intensity; AD-T: total sum of accelerations and decelerations; SHRZ: sum of heart rate zones; RPE: ratio of perceived physical effort; RPE Cog: ratio of perceived mental effort; sRPE: ratio of perceived physical effort x total duration of the session; sRPE Cog: ratio of perceived mental effort x total duration of the session.

The analyzed data came from the players’ daily routines follow-ups (see Figure 1). The following are the descriptive parameters of the tasks that could condition the cognitive load (Cárdenas et al., 2015): numerical ratio of players, available time, available space, task degrees of freedom and competitive stimulus. During each session, the objective external (Stojanović et al., 2021), internal (Scanlan et al., 2017) and cognitive loads (Zamora et al., 2020) were recorded using Polar Team Pro^1^ (Kempele, Finland) technology (Boyd et al., 2013). After each training session, the perceived subjective internal (Piedra et al., 2020) and cognitive loads (Fuster et al., 2021) were recorded. Data recorded from rehabilitation, strength sessions, individual sessions, warm-ups and matches were excluded. Only all training sessions that took place within this period were analyzed.

As a pilot period, a three-week trial was established, in order to get acquainted with the measuring devices and questionnaires, as well as the use of descriptive parameters for the tasks. In no case did the data collection affect the training dynamics. Incomplete records were excluded from the sample.

### Sessions and type of sessions

Training sessions were classified as MD + 1 (1 day after match day), MD + 2 (2 days after match day), MD-4 (4 days before match day), MD-3 (3 days before match day), MD-2 (2 days before match) and MD-1 (1 day before match day) according to their distance between one match and the next one (Oliva-Lozano et al., 2021a). In this study, MD + 1 days were not considered, since they were days off. The lowest intensity values occur on MD + 1, and they progressively increase until MD-3 (Ramos-Cano et al., 2022). Following this increase in load, a decrease can be observed until match day (Fernández et al., 2021). This microcycles structure is proposed as a strategy to alleviate accumulated fatigue and promote optimal performance levels on match days (Akenhead et al., 2016).

### Task descriptions

The defining variables of the tasks were recorded during their execution following observational methodology. As proposed by Schelling and Torres-Ronda (2013), the sessions’ tasks were classified according to their level of specificity by differentiating four orientations (general, directed, specific and competitive). The general orientation is associated with generic endurance tasks; the directed orientation refers to those specific tasks without opposition (1 × 0, 2 × 0, 3 × 0); the specific orientation hosts all small-sided games (SSG) situations (1 × 1, 2 × 2, 2 × X, 3 × 3, 3 × X, and 4 × X); and the competitive orientation refers to real or simulated game tasks (4 × 4, 5 × X, and 5 × 5).

On the other hand, the task classification proposed by Cárdenas et al. (2015) was used to differentiate the parameters that condition the degree of uncertainty in the tasks. They were described according to the numerical ratio of athletes interacting at the same time in a given space, i.e., the number of players involved in the attack and the defense; the dimension of the playing space, a quarter, half or the full court; the available time to solve the tasks as an indicator of time pressure, making distinctions between those exercises that had a restriction and those that did not; the degrees of freedom of the task, i.e., open tasks (the decision-making was free), semi-open (they could be A or B) and closed (there was no decision-making, there was a specific answer) (Farrow et al., 2008); and whether there was a competitive stimulus, separating those tasks that were competitive (outcome-dependent) from those that were not.

Each of the tasks was recorded and analyzed separately with the Polar Team Pro software. The variables analyzed in each task were determined to be reliable, showing the following Guttman Lambda 6 (G6) interval values for IL (G6 95% CI = 0.86–0.89), EL (G6 95% CI = 0.960.97) and CL (G6 95% CI = 0.570.62).

### Equipment and analyzed variables

#### External load variables

We analyzed the acceleration and deceleration data of the players as an objective indicator of external load (Piedra et al., 2021). The data was recorded using the indoor system of the Polar Team Pro devices, which have a sampling frequency of 200 Hz. Each player always used the same sensor. Accelerations and decelerations were classified into four levels according to their intensity, considering A1 (0.5 m/s^2^ – 0.99 m/s^2^), A2 (1 m/s^2^ – 1.99 m/s^2^), D1 (−0.5 m/s^2^ – -0.99 m/s^2^), D2 (−1 m/s^2^ – -1.99 m/s^2^) as low intensity, and A3 (2 m/s^2^ – 2.99 m/s^2^), A4 (> 3 m/s^2^), D3 (−2 m/s^2^ – -2.99 m/s^2^), D4 (> − 3 m/s^2^) as high intensity. The total high intensity accelerations (HI-A) are the sum of A3 and A4, and the total high intensity decelerations (HI-D), the sum of D3 and D4. The total amount of high intensity actions (HI-T) is the sum of HI-A and HI-D. Also analyzed is the total sum of accelerations and decelerations (AD-T).

#### Internal load variables

We analyzed the players’ Heart Rate (HR) data as an objective indicator of internal load (Berkelmans et al., 2018). The summed heart rate zone (SHRZ) model was used as an internal load indicator based on HR, determined by multiplying the HR zones weighted by the duration (min) elapsed in each zone as follows (Edwards, 1993): Zone 1 = 50 to 60% HR max.; Zone 2 = 60 to 70% HR max.; Zone 3 = 70 to 80% HR max.; Zone 4 = 80 to 90% HR max.; and Zone 5 = 90 to 100% HR max. The SHRZ model has been frequently used to quantify IL in basketball (Fox et al., 2018) and has been shown to be sensitive to load changes during training periods (Scanlan et al., 2014).

To measure the subjective Internal Load as a variable (Piedra et al., 2020) athletes were asked to complete a questionnaire 30 min after the end of each training session, reporting in a single score their perceived physical exertion throughout the session (RPE) on a CR10 scale, adapted from Foster et al. (2001). Players were instructed to provide the data honestly and individually. They were also informed that no negative consequences would arise in relation to their responses. The perceived training load (sRPE), presented in arbitrary units (a.u.) to quantify the internal load, was calculated by multiplying the recorded RPE by the respective session duration (min).

#### Cognitive load variables

Continuously recorded heart rate variability (HRV) (Ramos-Campo et al., 2017) was used as an objective variable to assess cognitive load (Fuster et al., 2021). We used the variable RMSSD, which represents the square root of the mean of the sum of the squared differences of all RR intervals (time in milliseconds between consecutive heartbeats). RMSSD reflects the heartbeat-to-heartbeat variance in the heart rate and shows the short-term variability of the HR (Shaffer and Ginsberg, 2017). This time variable is related to the parasympathetic system and is defined as a global indicator of the psychophysiological fatigue of the athlete (Schmitt et al., 2015). HRV has been shown to be a variable sensitive to cognitive load demands (Luque-Casado et al., 2016). For instance, Jin et al. (2022) demonstrated that HRV gradually decreases as the number of players involved in the basketball drills. Thus, a higher specificity is related to a lower HRV of the players during the tasks (Jin et al., 2022). Consequently, Portes et al. (2024) determine HRV as a reliable tool to monitor the psychophysiological state of a basketball player.

To measure the subjective Cognitive Load as a variable (Fuster et al., 2021), as with the RPE, athletes were asked to complete a questionnaire 30 min after the end of each session, reporting in a single score their perceived mental effort throughout the session (RPE Cog) on a CR10 scale, adapted from Foster et al. (2001). The perceived training load (sRPE Cog), presented in arbitrary units to quantify cognitive load, was calculated by multiplying the recorded RPE Cog by the respective session duration (min) (Romero-Moraleda et al., 2023).

### Statistical analysis

The statistical analysis was performed with JASP software version 0.17.2.1 Intel (Jasp Team, Amsterdam, Netherlands). The tasks’ descriptive data is presented as percentages and load variables as an average ± standard deviation (SD). A descriptive analysis of central tendency was performed, and through the Shapiro–Wilk test, we determined the non-normality of the sample. The Kruskal-Wallis test was used to evaluate the effects of the different sessions with their respective tasks (independent variables) over the CL, EL and IL variables (dependent variables). With this goal in mind, we used the RPE, RPE Cog, sRPE, sRPE Cog, HI-T, AD-T, HRV and SHRZ variables. In turn, a Dunn-Bonferroni *post hoc* test was performed. The reliability of intrasession measures was determined through the Guttman’s Lambda 6 test, with confidence intervals of 95% (Oosterwijk et al., 2016).

Furthermore, with the intention of determining the relationship between the load variables, we performed the Pearson’s Chi-square test. The degree of association is assessed through the Goodman-Kruskal’s Gamma test. Gamma correlations with absolute values under 0.3 are considered negligible (Mukaka, 2012). Finally, to explore the possible distribution of the different assumed loadings in homogeneous groups, a cluster analysis was performed. We grouped the variables into clusters through the K-Mean method. Once the clusters were established, possible differences were determined through an ANOVA analysis (Izquierdo et al., 2019). The significance level was set at *p* < 0.05.

## Results

A total of 11 microcycles, 42 sessions and 109 valid exercises were recorded. These sessions are distributed in 4 MD + 2 sessions, 9 MD-4 sessions, 12 MD-3 sessions, 12 MD-2 sessions and 5 MD-1 sessions. The difference between sessions registers (e.g., only 4 MD + 2 sessions were recorded) is due to factors beyond the researcher’s control (failure of the recording system, loss of data, etc.) or because training sessions were carried out in other facilities for logistic and scheduling reasons.

**Figure 2:**
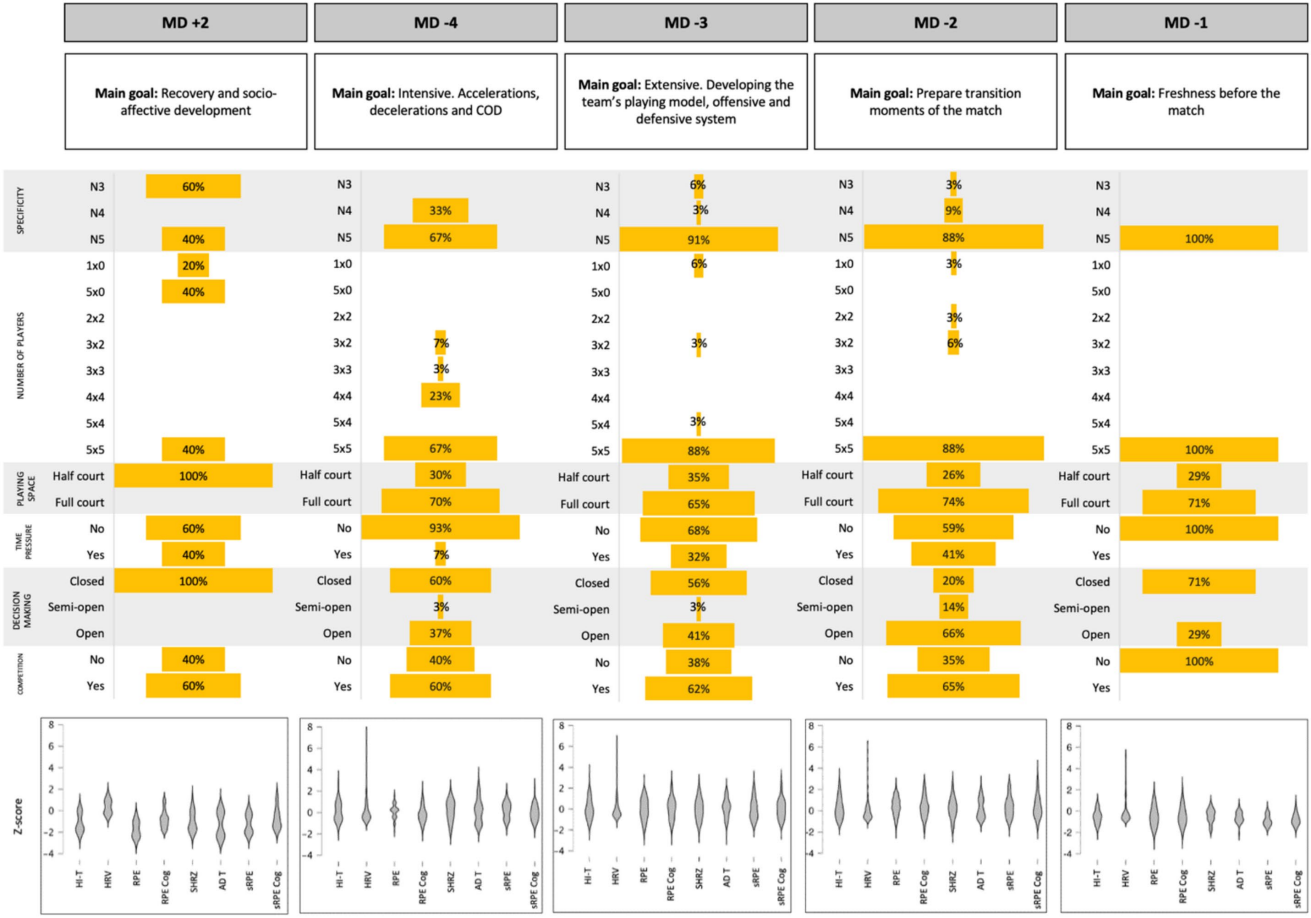
General description of the standard competitive microcycle. Presentation of the main objectives of each session, the descriptive variables of the tasks expressed in percentage and the loading variables in *z*-score format. RPE: ratio of perceived physical effort; RPE Cog: ratio of perceived mental effort; sRPE: ratio of perceived physical effort × total duration of the session; sRPE Cog: ratio of perceived mental effort × total duration of the session; SHRZ: sum of heart rate zones; HI-T: sum of actions at high intensity; AD-T: total sum of accelerations and decelerations; HRV: RMSSD parameter of heart rate variability; MD ± x: x days after (+) or before (−) the match day.

Tables 1, 2 show the quantitative distributions of the CL, EL and IL, as well as the descriptive variables of the tasks that make up each session, classified according to the distance from the match, following the structured microcycle methodological model proposed by Tarragó et al. (2019). The MD-2 training session shows the highest values for all the variables described (RPE = 6.4 ± 0.7; RPE Cog = 4.9 ± 0.9; sRPE = 309.7 ± 97.8; sRPE Cog = 240.4 ± 86.1; SHRZ = 122.1 ± 28.8; HI-T = 131.7 ± 29.7; AD-T = 896.6 ± 204.3), except for HRV, being session MD + 2 the one with the highest values (HRV = 20.5 ± 1.0). Figure 2 shows a general description of all the study’s goals: the tasks’ descriptive data percentages and the normalized values with the load variables’ z-score by type of session. Session MD + 2 differs the most from the rest, showing the lowest uncertainty values in the training tasks and the lowest demands on Cl, EL and IL. Considering the specificity of the tasks, we observed a clear increasing trend in the use of competitive tasks as the day of competition approaches, as well as a decrease in the use of directed (mainly) and specific tasks.

**Table 1:** Descriptive statistics (mean ± standard deviation) of all loading variables in relation to match day.

Load variables	MD +2	MD -4	MD -3	MD -2	MD -1
RPE (0–10 scale)	3,0 ± 1,4	5,6 ± 0,9	5,6 ± 1,3	6,4 ± 0,7	4,6 ± 0,3
RPE Cog (0–10 scale)	2,9 ± 1,2	3,9 ± 0,6	4,3 ± 1,2	4,9 ± 0,9	3,7 ± 0,3
sRPE (a.u.)	132,5 ± 129	271,5 ± 92,8	268 ± 103,4	309,7 ± 97,8	183,5 ± 32,8
SRPE Cog (a.u.)	124,5 ± 118,0	186,4 ± 59,1	204,4 ± 74,6	240,4 ± 86,1	127,5 ± 22,2
SHRZ (a.u.)	62,9 ± 47,4	111,1 ± 33,7	111,7 ± 31,0	122,1 ± 28,8	85,3 ± 12,1
HI - T (a.u.)	70,5 ± 42,0	117,6 ± 30,7	121,7 ± 31,8	131,7 ± 29,7	92,1 ± 9,7
AD - T (a.u.)	575,9 ± 349,3	849,8 ± 271,5	887,7 ± 180,7	896,6 ± 204,3	736,1 ± 115,5
HRV (RMSSD)	20,5 ± 1,0	18,8 ± 7,0	15,2 ± 2,3	15,1 ± 3,7	15,8 ± 3,8

RPE: ratio of perceived physical effort; RPE Cog: ratio of perceived mental effort; sRPE: ratio of perceived physical effort × total duration of the session; sRPE Cog: ratio of perceived mental effort × total duration of the session; SHRZ: sum of heart rate zones; HI-T: sum of actions at high intensity; AD-T: total sum of accelerations and decelerations; HRV: RMSSD parameter of heart rate variability; MD ± x: x days after (+) or before (−) the match day.

**Table 2:** Descriptive analysis (expressed as a percentage) of the training tasks in relation to the match day.

Descriptive variables		MD +2	MD -4	MD -3	MD -2	MD -1
Specificity	N3	60%		6%	3%	
	N4		33%	3%	9%	
	N5	40%	67%	91%	88%	100%
Number of players	1 × 0	20%		6%	3%	
	5 × 0	40%				
	2 × 2				3%	
	3 × 2		7%	3%	6%	
	3 × 3		3%			
	4 × 4		23%			
	5 × 4			3%		
	5 × 5	40%	67%	88%	88%	100%
Playing space	Half court	100%	30%	35%	26%	29%
	Full court		70%	65%	74%	71%
Time pressure	No	60%	93%	68%	59%	100%
	Yes	40%	7%	32%	41%	
Decision making	Closed	100%	60%	56%	20%	71%
	Semi-open		3%	3%	14%	
	Open		37%	41%	66%	29%
Competition	No	40%	40%	38%	35%	100%
	Yes	60%	60%	62%	65%	

N3: Level 3 approach to specificity; N4: Level 4 approach to specificity; N5: Level 5 approach to specificity; Closed decision making; no decision making; Semi-open decision making: two decision options; Open decision making: freedom of decision; MD ± x: x days after (+) or before (−) the match day.

The Kruskal-Wallis’s test showed significant differences for all load variables RPE, RPE Cog, sRPE, sRPE Cog, SHRZ, HI-T, AD-T (*H* = 37.505–73.649; df = 4; *p* = <0.001), except for HRV (*H* = 14.571; df = 4; *p* = 0.006), by session type. In turn, the *post hoc* test also determined that these differences were present between each of the variables for the same team (*z*: −6.053 to 7.788; wi: 40.250 to 254.826; wj: 94.490 to 254.826; pBonf: <0.001). The post hoc showed significant differences between MD + 2 and the rest of the sessions in relation to RPE. Regarding the variables sRPE, RPE Cog, sRPE Cog, SHRZ, HI-T and TOTAL AD, significant differences were observed between MD + 2 and MD-4, and between MD-3 and MD-2.

On the other hand, the Chi-square test, using the Goodman-Kruskal Gamma as the association value, showed moderate and high associations between the descriptive variables of the task and the RPE, considering HRZ as a cognitive load variable (*p* < 0.01; G 0.522), considering HI-T as an external load variable (*p* < 0.01; G 0.46) and considering SHRZ as an internal load variable (*p* < 0.01; G 0.468). These were low among the task descriptive variables and the RPE Cog. Finally, the cluster grouping using the K-Mean method showed 10 distinct groups (*R*^2^ = 0.802; Silouette = 0.19) using the SHRZ, HI-T and RPE Cog variables.

## Discussion

This study allowed us to observe the dynamics of the training loads, in its cognitive, external and internal dimensions, with the goal of monitoring the athletes’ performance level in each specific training session. The descriptive analysis of the dynamics of loads revealed significant differences for the different metrics of CL, EL and IL between the sessions of the structured microcycle, in which MD + 2 and MD-1 showed the lowest demands. This profile may be associated with the recovery, accumulation and optimization processes within the same competitive microcycle. Finally, moderate relationships were detected between the variables of CL, EL and IL and the descriptive variables of the task, suggesting the interrelation of the different loads.

The dynamics of CL, EL and IL throughout the microcycle, and depending on the type of session, can provide information regarding the degree of task uncertainty (Cárdenas et al., 2015). An increasing trend was observed between MD + 2 to MD-1 in task specificity, number of players and space (see Figure 2); on the other hand, the highest time pressure, decision-making and competitive stimulus are found in sessions MD-3 and MD-2 presenting a parabolic trend throughout the microcycle. It should be noted that the dynamics of cognitive, external and internal loads show similar trends, with the central part of the week having the highest load demand. These results could be in line with the approach of Fuster et al. (2021), in which CL, EL and IL are defined as separate interdependent constructs, in which the manipulation of one of these will cause changes in the others. Other studies have also shown parabola-shaped distributions in the dynamics of competitive microcycle loads (Ammann et al., 2023; Akenhead et al., 2016; Fernández et al., 2021). On the other hand, other authors observed a load reduction throughout the microcycle, but with a downward trend (Stevens et al., 2017; Vachon et al., 2021; Garcia et al., 2022a). The obtained results follow in line with previous studies, presenting the tendency to reduce the loading parameters in the days prior to a competition, following a strategy of gradual reduction and to optimize match performance (Martín-García et al., 2018).

Significant differences were also observed between training sessions. The training contents prescribed in the MD + 2 sessions, which aim at active recovery and the socio-affective development of the team, could explain the lowest values of the load variables, since it’s the session with the lowest specificity and prescribed load. The HRV being highest on MD + 2 sessions could be an indicator of the extent of the individual recovery process (Moreno et al., 2015), following the proposal of Querido et al. (2023), in which MD + 2 sessions were included as an essential part of the physiological and psychosocial recovery process of the athletes. Furthermore, these results are also aligned with those obtained by Jin et al. (2022), in which they describe a gradual decrease in HRV as the number of players participating in basketball tasks increases, attending to the increase in specificity. On the other hand, no significant differences were found between MD-4, MD-3 and MD-2, with these sessions having the highest prescribed loads; but there were significant differences in RPE Cog between MD-4 and MD-2, with a higher value in MD-2. Such differences could be explained due to changes in the descriptive variables of the tasks, where MD-2 is the session with the highest specificity and uncertainty, causing an increase in CL (Cárdenas et al., 2015). In this regard, Tarragó et al. (2019) propose a structured microcycle based on the evolution of specificity and variability to optimize match performance. Furthermore, significant differences in RPE and sRPE were also found between the MD-3 and MD-2 sessions, the latter being the ones with the highest results. Other basketball research (Vázquez-Guerrero et al., 2018) also suggests that the greater the space used on the court during training exercises, the greater the EL imposed on the players, with our research backing up this claim; this increase in EL causes, among other things, an increase in RPE and sRPE (Scanlan et al., 2014). Finally, a significant decrease in all the studied variables was observed in the MD-1 sessions. This reduction of CL, EL and IL in MD-1 training sessions denotes how the aim of planification is the gradual reduction of loads (Svilar et al., 2019) to optimize on competition days in team sports (Akenhead et al., 2016; Illa et al., 2020; Martín-García et al., 2018; Vachon et al., 2021).

Finally, we analyzed the common variability between CL, EL and IL. The moderate and high relationships found amongst the tasks’ descriptive variables and RPE, accounting for HRZ as a cognitive load variable (*p* < 0.01; G 0.522), accounting for HI-T as an external load variable (*p* < 0.01; G 0.46) and accounting for SHRZ as an internal load variable (*p* < 0.01; G 0.468), show a dependency between CL, EL and IL variables, thus reinforcing Fuster et al. (2021)‘s proposal, in which CL, EL and IL are defined as separate interdependent constructs. The results obtained by grouping the SHRZ, HI-T and RPE Cog variables in clusters through the K-Mean method could help to interpret how the different constructs (CL, EL and IL) behave and relate to each other in the same tasks, given that intervening one of them may affect the others. These ten clusters may correspond to the values of the RPE Cog’s Borg scale and may offer a variable that would allow us to jointly manage others such as SHRZ and HI-T (Perrey, 2022).

This study has certain limitations. First, all incomplete records were excluded from the study because of missing variables for at least one of the CL, EL and IL measures. Such missing values could be explained by errors with the recording devices or because the players did not respond to the respective RPE and RPE Cog questionnaires. In addition, the structures of the microcycles were not stable, being altered according to the competitive calendar, causing an imbalance between the number of sessions recorded. These situations are characteristic of competitive environments such as the one described here, due to a congestion of the competitive calendar, an increase in training sessions and a higher frequency of travel (Calleja-Gonzalez et al., 2020). Precisely because of these particularities of the competition, only part of the season was recorded instead of the whole season. These results are specific and applicable to this particular group, but the methodology can be extrapolated to other groups or teams. It would be interesting to be able to expand the number of participating teams in order to generalize the results to other populations or competitive levels. Nevertheless, and to the authors’ knowledge, this is the first study that carefully describes the dynamics of CL, EL and IL during the competitive microcycle in professional sport.

## Conclusion

In conclusion, considering the obtained results, the load dynamics showed an increase in uncertainty throughout the microcycle, from less to more specific, and a load distribution where MD + 2 and MD-1 had the lowest values and MD-4, MD-3 and MD-2 the highest. Significant differences (*p* < 0.01) between sessions were found for all the analyzed variables. Possible relationships between the CL, EL and IL metrics were also determined. This study shows the reality of a professional team, where the distance from the next match determines the dynamics of the workload, promoting an increase in uncertainty and specificity throughout the microcycle, thus causing an increase in cognitive load.

### Practical applications

This study may establish a starting point for future research on load control in team sports, integrating cognitive load as one of the three central pillars, together with external and internal loads. Considering the possible relationship between the variables CL, EL and IL, it would be possible to jointly manage load demand by intervening in at least one of them. Isolated assessment of CL, EL and IL provides information about the stimulus delivered, the processing or the response, without examining the inherent relationship.

The current reality of high-performance teams has a very saturated competitive calendar and a high volume of training sessions, so managing the cognitive load throughout the microcycle, reducing the specificity at the beginning of it, will promote better adaptations in both conditional and emotional aspects. A holistic view would allow a deeper understanding of load distribution, providing information applicable to staff members (coaches, trainers, physical trainers, physiotherapists, psychologists, and physicians).

### Future directions of research

Sport needs applied science to keep improving. Understanding the effect of training loads, from this new perspective, will allow us to continue advancing in the field of performance optimization and injury prevention. An extension of this study is proposed, taking into account potential confounding factors such as fatigue, sleep, travel, or the phase of the menstrual cycle. In the same way it would be very interesting to make a longitudinal study of a whole competitive season for a greater applied relevance and deepen the evolution of load dynamics throughout the season, as well as a relational analysis with indicators of performance or injury risk to expand knowledge in this field.

## Data Availability

The original contributions presented in the study are included in the article/supplementary material, further inquiries can be directed to the corresponding author/s.

## Footnotes

^1^ https://teampro.polar.com
